# Single-molecule localisation microscopy: accounting for chance co-localisation between foci in bacterial cells

**DOI:** 10.1007/s00249-021-01555-z

**Published:** 2021-06-19

**Authors:** Christoffer Åberg, Andrew Robinson

**Affiliations:** 1 Groningen Research Institute of Pharmacy, University of Groningen, Antonius Deusinglaan 1, 9713AV Groningen, The Netherlands; 2 Molecular Horizons Institute and School of Chemistry and Molecular Bioscience, University of Wollongong, Wollongong, NSW 2522 Australia; 3 Illawarra Health and Medical Research Institute, Wollongong, NSW 2522 Australia

**Keywords:** Single-molecule experiments, Fluorescence microscopy, Co-localisation, Radial distribution function, Pair distribution function, Bacterial cells

## Abstract

Using single-molecule fluorescence microscopes, individual biomolecules can be observed within live bacterial cells. Using differently coloured probes, physical associations between two different molecular species can be assessed through co-localisation measurements. However, bacterial cells are finite and small (~ 1 μm) relative to the resolution limit of optical microscopes (~ 0.25 μm). Furthermore, the images produced by optical microscopes are typically two-dimensional projections of three-dimensional objects. These limitations mean that a certain proportion of object pairs (molecules) will inevitably be assigned as being co-localised, even when they are distant at molecular distance scales (nm). What is this proportion? Here, we attack this problem, theoretically and computationally, by creating a model of the co-localisation expected purely due to chance. We thus consider a bacterial cell wherein objects are distributed at random and evaluate the co-localisation in a fashion that emulates an experimental analysis. We consider simplified geometries where we can most transparently investigate the effect of a finite size of the cell and the effect of probing a three-dimensional cell in only two dimensions. Coupling theory to simulations, we also study the co-localisation expected due to chance using parameters relevant to bacterial cells. Overall, we show that the co-localisation expected purely due to chance can be quite substantial and describe the parameters that it depends upon.

**Supplementary Information:**

The online version contains supplementary material available at 10.1007/s00249-021-01555-z.

## Introduction

Single-molecule localisation microscopy (SMLM) is most widely known as the basis for the super-resolution microscopy techniques PALM (Photo-Activated Localization Microscopy) and STORM (Stochastic Optical Reconstruction Microscopy) (Schermelleh et al. 2019). By detecting fluorescence signals emanating from individual fluorescent proteins or organic dyes, these techniques allow the spatial positions of biomolecules within cells to be mapped at high resolution. However, SMLM is also useful for monitoring the spatiotemporal dynamics of biomolecules within cells (Kapanidis et al. 2018). Commonly, SMLM is used to monitor the binding and dissociation of fluorescently labelled small biomolecules, such as proteins, to larger structures, such as chromosomes or cell membranes, within bacterial cells. Such measurements make use of changes in the diffusive motion of the smaller molecule that occur upon binding to the larger, more static, structure. Protein molecules diffuse through the cytosol of bacterial cells with a diffusion coefficient *D* ~ 10 μm^2^ s^−1^ (Schavemaker et al. 2017). Relative to typical image exposure times (10–100 ms), these molecules move so quickly that the fluorescence signal they produce spreads evenly over the entire bacterial cell (Yu et al. 2006). If, as part of its normal function, an individual protein molecule binds to the chromosome, or other large structure, its diffusion rate decreases by many orders of magnitude (*D* ~ 10^–5^ μm^2^ s^−1^ for chromosome-associated proteins) (Yu et al. 2006; Elf et al. 2007; Reyes-Lamothe et al. 2008). As a result, that fluorescent protein molecule presents in the microscopy image as a relatively static focus (Yu et al. 2006; Elf et al. 2007; Kapanidis et al. 2018). A large number of studies have exploited this phenomenon, known as detection-by-localisation, to study the binding of proteins to the bacterial chromosome, cell membrane, and other large structures (Kapanidis et al. 2018; Joseph and Badrinarayanan 2020; Lagage and Uphoff 2020).

A series of SMLM studies have used two-colour imaging to measure the extent of co-localisation between foci formed by different types of molecules within cells, with a view towards inferring molecular associations between those molecules (Vojnovic et al. 2019). In some instances, the proportion of foci that are measured to be co-localised has been quite modest. For example, in a study carried out by the Robinson group, only 5–10% of DNA polymerase IV foci co-localised with replication fork markers (Henrikus et al. 2018). In all cases, but especially when co-localisation is modest, it is important to consider the ‘baseline’ level of co-localisation that is expected to occur by chance. By this, we refer to molecules that are not physically associated with each other, but spatially overlap in microscope images due to the small dimensions of the cell that confines them and the limited resolution of optical microscopes.

There are many studies that have thoroughly explored the significance of co-localising signals in microscopy images, for instance using Pearson’s correlation coefficient (Manders et al. 1992) or Manders’ overlap coefficients (Manders et al. 1993). However, only one study (Helmuth et al. 2010) has explored the form of measurement carried out in multi-colour SMLM measurements (Dunn et al. 2011). Thus, Helmuth et al*.* (2010) has provided a general statistical inference framework that generalises co-localisation analysis to one of extracting interactions, testing its utility on virus trafficking in human cells. The present work is complementary to Helmuth et al*.* (2010) adding: (i) an *analytical* calculation of the distribution of distances between object (focus) pairs; and (ii) results representative of bacterial cells, extracted from simulations in which objects (foci) are placed at random.

Specifically, our analytical approach starts with the simplest situation and progressively add complicating features to the problem to understand the effect of each aspect. To evaluate the co-localisation expected due to chance, one could in principle simply perform numerical simulations where one places a certain number of objects within the volume of interest, and subsequently evaluates the co-localisation in a manner that emulates the procedure done experimentally. However, we choose the analytical approach, to gain generalisable information. Thus, we start by defining what we actually mean by co-localisation due to chance and discussing the limitations. Subsequently, we introduce the concept of distribution of distances within the volume, from which we can readily calculate the co-localisation due to chance of two objects. Obviously, limiting ourselves to two objects is not reasonable, but initially focussing on only two objects allows us to most clearly bring out several features. The derivations are based upon a similar derivation for the distribution of projected distances for objects on the surface of a sphere (Kelly et al. 2015) and are mostly relegated to Online Resource 1 to keep the main text brief. We consider the distribution of distances in three simple geometries which allows explicit analytical solutions. We start with the circle, from which we can clearly derive the effect of a finite volume. We next consider the effect of not being able to sample in the axial direction, by considering only distances in projected coordinates, for a cylinder and for a sphere. We then lift the limitation on two objects and examine the combinatorial effects that must be included when considering the co-localisation of an arbitrary number of objects. Finally, with this background we exemplify the co-localisation expected due to chance using realistic parameters for bacteria.

## Results and discussion

To create a model of co-localisation due to chance, we consider two types of objects, type A and type B, which distribute at random within a certain volume. We are specifically interested in co-localisation measurements in bacterial cells, but since the majority of results are fairly general, we will use a neutral phrasing. Thus, the objects could be single molecules, oligomers, other molecular complexes, organelles, other vesicles, probe particles or possibly something else. Similarly, the volume could be a cell, but it could also be a more limited part of a cell (e.g., the nucleus of a eukaryotic cell).

### Definition of random distribution

We start by defining exactly what we mean by a random distribution. We will assume that each of the two types of objects in question distribute within the cell in such a way that the probability of being in a certain position is uniform inside the cell, independently of the position of the other objects. Obviously, if a certain type of object only distribute within a smaller volume of the cell (e.g., the nucleus in a eukaryotic cell) then the space to be considered is not the full cell, but the smaller volume (nucleus). It may also be that a certain object distributes within the full volume, but not uniformly (e.g., more often in one side of the cell than the other). This could be an important problem, but it is difficult to imagine that it could be resolved with some generality, so we will leave such problems to be dealt with on a case-by-case basis.

Within our assumption, we must distinguish independent distribution with respect to objects of the same type and with respect to objects of the other type. The distribution with respect to objects of the other type is in reality not necessarily independent. Indeed, that is the purpose of evaluating the co-localisation in the first place. Thus, we must essentially make this assumption by definition. The assumption that an object distributes independently of the position of the other objects *of the same type* also seems natural. Of course, interactions may cause this not to be the case. However, if the interaction is weak, then an independent distribution will be a good approximation; conversely, if the interaction is strong, the object will oligomerise and we may assume independent distribution for the oligomer instead. We envisage that an intermediate interaction, which affects the distribution but nevertheless does not cause oligomerisation, would not occur often in practice. There remains the rather delicate possibility that the distribution with respect to the other objects of the same species is different in the presence of the other species, without the two species co-localising proper. In this case, the assumption of independence would underestimate the co-localisation expected due to chance. However, one may view it rather appropriate that such a circumstance shows a co-localisation higher than expected.

A more general shortcoming behind assuming independent positioning is that in reality two objects cannot overlap and so their distribution is by necessity not independent. Nevertheless, objects of interest are often small enough (especially considering the resolution limit implied by experimental set-ups) that the correction due to finite size of the objects is small.

It is important to have these caveats in mind. Overall, though, our assumption that objects distribute uniformly and independently within the space we consider seems rather natural for many cases of practical interest.

### Distribution of distances

To gain some insight into the issues, we will start by considering the distribution of distances within a given volume. With distribution of distances, we here mean the distance between two arbitrary points within the volume, averaged over the position of those two points. In other words, the distribution of distances, d*n*(*ρ*), is the probability that two objects, if placed at random and independently within the volume, are separated a certain distance, *ρ*. It is normalised such that integration over all distances (from 0 to the largest distance within the volume) is unity. Phrased in this latter way, it is clear that the distribution of distances is rather intimately related to the co-localisation of two objects, when the two objects distribute randomly and independently. In fact, the probability of having two objects co-localised is simply given by the integral

1 $${\int }_{0}^{\xi }\text{d}n\left(\rho \right),$$

where *ξ* is the distance within which we consider the two objects to be co-localised. *ξ* will be given by details of the experimental set-up, including the optical diffraction limit. We should stress that this is, indeed, the co-localisation between *two* objects. However, for more than two objects, combinatorial and many-body effects also come into play. This will be discussed in a later section.

When considering the distribution of distances, we have implicitly ignored the actual position of an object. That is, if an object is, say, situated close the edge of the volume considered, then the possible distances is different compared to an object situated in the centre of the volume. Thus, we could in principle consider a distribution of distances that depends on the position within the volume. By extension, the co-localisation expected due to chance would be different depending upon the position within the volume. This can be done, at least with simulations (Helmuth et al. 2010), but for analytical and general results, it quickly becomes intractable. Consequently, we will ignore the extra information that is in principle there in the position.

We also mention that, save for the choice of normalisation and division by *ρ*^2^, the distribution of distances has a long history of usage within statistical mechanics where it is known as the radial distribution function, pair distribution function or simply by its conventional designation *g*(*r*) (Widom 2002; Binder and Kob 2005; Hansen and McDonald 2013). From this perspective, our assumption of random and independent distribution within the volume is equivalent to the assumption of an ideal gas. This connection may be useful for interpretation. However, there are some clear differences compared to the usage in statistical mechanics: first, we are interested in objects confined within a finite volume (e.g., a cell) rather than the infinitely large systems implied by the thermodynamic limit. This is particularly pertinent when the distance used to define co-localisation is not vastly different (that is, not several orders of magnitude smaller) than the size of the space considered. The correction due to finite size comes out of the mathematics quite clearly, as will transpire below. A related difference is that the distribution of distances is neither homogeneous nor isotropic. This is an aspect we just argued was impractical for comparison to experiments.

### Distribution of distances within a circle

**Fig. 1:**
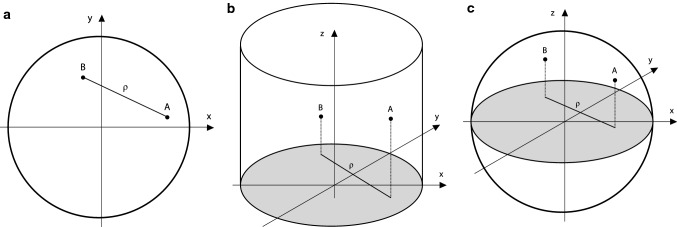
Distribution of distances for the three different situations considered analytically. A and B signify two arbitrary points and *ρ* the distance between them. **a** Circle. **b** Cylinder, where the distance considered is the distance projected onto the *xy* plane. **c** Sphere, where the distance considered is the distance projected onto the equatorial plane

We start by considering the distribution of distances within a circle. We reiterate that this will not give an answer to the question of what co-localisation is expected due to chance in general, but merely forms a stepping stone towards it. As discussed, our underlying assumption is that objects distribute by chance uniformly and independently within the circle. Based on this assumption, we can then calculate the distribution of inter-object distances, *ρ*, by positioning two points, A and B, within the circle. We may position A anywhere and we position B at a distance *ρ* from A (Fig. 1a). Subsequently, we integrate over all possible positions for A and B. The calculation is facilitated using the high degree of symmetry of a circle. For brevity, we do not reproduce the full derivation here (see Online Resource 1, “Derivation of distribution of distances within a circle” section) but simply quote the final result. Thus, the distribution of distances is given by

2 $$ {\text{d}}n\left( \rho  \right) = \frac{2}{{R^{4} }}\rho {\text{d}}\rho \left( {R^{2}  - {\text{rect}}_{{\left[ {R,2R} \right]}} \left( \rho  \right)\left( {R - \rho } \right)^{2}  - \frac{2}{\pi }\mathop \smallint \limits_{{x_{{\text{A}}}  = {\text{max}}\left( {R - \rho ,\rho  - R} \right)}}^{{x_{{\text{A}}}  = R}} x_{{\text{A}}} {\text{arccos}}\left( {\frac{{R^{2}  - x_{{\text{A}}}^{2}  - \rho ^{2} }}{{2x_{{\text{A}}} \rho }}} \right){\text{d}}x_{{\text{A}}} } \right), $$

where *R* is the radius of the circle. This distribution has been normalised such that the integral of d*n*(ρ) over all distances (0 to 2*R*) is unity.

To show the physical meaning of this distribution, it is useful to consider it in the limit that *ρ*/*R* is small, that is, in the limit that the distances we consider are much smaller than the radius of the circle. We then expect that the circle will be of less importance. To first order, we remain with only the first term within the (outmost) parentheses of Eq. (2), i.e., the result

3 $$\text{d}n\left(\rho \right)=\frac{2}{{R}^{2}}\rho \mathrm{d}\rho +\cdots .$$

This can be argued to agree with results from statistical mechanics (see Online Resource 1), as it should, because in that case one indeed assumes large (infinite) systems.

While this distribution is only the distribution of distances, not co-localisation, we can nevertheless use it for the (very) special case of evaluating the co-localisation due to chance of exactly two objects. In other words, we ask the question what the probability is that two objects inside the circle are within a distance *ξ* apart. A simple integration of Eq. (3) then shows that this probability is (*ξ*/*R*)^2^, i.e., equivalent to the area of a circle of radius *ξ* over the area of the full circle, *R*. We reiterate that this remains true only for two objects. Still, as long as the distances considered are small compared to the size of the circle, the probability that two objects are within a certain distance from each other is simply given by the ratio of the area of overlap to the total area. This is certainly a useful first approximation and one that could be considered rather intuitive.

We continue with the next order approximation which would be applicable to distances smaller than the radius of the circle, but not negligibly so. Equation (2) then reads

4 $$\text{d}n\left(\rho \right)=\frac{2}{{R}^{3}}\rho \mathrm{d}\rho \left(R-\frac{2}{\pi }\rho +\cdots \right).$$

Again illustrating the consequences of this result for the co-localisation of two objects, we integrate Eq. (4) to find that the probability that two objects inside the circle are co-localised is

5 $${\left(\frac{\xi }{R}\right)}^{2}\left(1-\frac{4}{3\pi }\frac{\xi }{R}\right).$$

If we compare this result to the first-order approximation [(*ξ*/*R*)^2^], we notice that the probability that two objects are close to each other is now lower. The reason is that an object that is close to the circle circumference is less likely have a neighbour within the circle, a situation that does not arise in free space and hence is a next-to-leading order effect.

### Distribution of distances within cylinder in projected coordinates

The example of a circle is of course very much simplified, but nevertheless allowed us to build some intuition on the effects we may expect in a more realistic setting. In that vein, when we now continue with understanding projection, we also consider a simplified situation. Thus, we consider a cylinder within which objects distribute uniformly and independently, but where we are only able to measure distances in the plane perpendicular to the cylinder axis (Fig. 1b). There is then a certain probability that two points far apart in the direction parallel to the cylinder axis, nevertheless appear to be close when only considering the projected distance. On the other hand, compared to the circle (above) there is also a decreased probability of being close because of the increased available space.

For a cylinder, these two effects exactly cancel (see Online Resource 1, “Distribution of distances within cylinder in projected coordinates” section for a brief argument), and the normalised distribution of (projected) distances is exactly the same as for the circle (Eq. 2). Note that this is not an asymptotic result for an infinitely long cylinder, but remains true for all lengths of the cylinder. Based on this observation, the same conclusions regarding the co-localisation of exactly two objects within the cylinder may be drawn as for a circle: the probability that two objects are within a (projected) distance *ξ* from each other is (*ξ*/*R*)^2^, if the distance is much smaller than the radius of the cylinder (the cylinder length is irrelevant); or somewhat smaller if the distance is more comparable to the cylinder radius (Eq. 5).

### Distribution of distances within sphere in projected coordinates

The same argument (Online Resource 1) that shows that there is no effect of the third dimension for a cylinder (above) can be generalised to show that there is necessarily no effect of projection for shapes that are uniform in the direction perpendicular to the projected plane. To nevertheless get an idea of the magnitude of the effect, we instead consider a different simplified system, namely a sphere where distances are assessed in the equatorial plane (Fig. 1c). The distribution of projected distances for objects that distribute uniformly and independently within the sphere is then given by (see Online Resource 1, “Derivation of distribution of distances within sphere in projected coordinates” section)

6 $$ {\text{d}}n\left( \rho  \right) = \frac{9}{{\pi R^{6} }}\rho {\text{d}}\rho \left( {{\text{rect}}_{{\left[ {0,R} \right]}} \left( \rho  \right)\mathop \smallint \limits_{{x_{{\text{A}}}  = 0}}^{{{\text{x}}_{{\text{A}}}  = R - \rho }} \mathop \smallint \limits_{{u =  - 1}}^{{u = 1}} f\left( {\rho ,x_{{\text{A}}} ,u} \right){\text{d}}x_{{\text{A}}} {\text{d}}u + \mathop \smallint \limits_{{x_{{\text{A}}}  = \max \left( {R - \rho ,\rho  - R} \right)}}^{{x_{{\text{A}}}  = R}} \mathop \smallint \limits_{{u =  - 1}}^{{u = \left( {R^{2}  - x_{{\text{A}}}^{2}  - \rho ^{2} } \right)/2{\text{x}}_{{\text{A}}} \rho }} f\left( {\rho ,x_{{\text{A}}} ,u} \right){\text{d}}x_{{\text{A}}} {\text{d}}u} \right) $$

in terms of the integrand.

$$f\left(\rho ,{x}_{\text{A}},u\right)=\sqrt{{R}^{2}-{x}_{\text{A}}^{2}}{x}_{\text{A}}\sqrt{\frac{{R}^{2}-{x}_{\text{A}}^{2}-{\rho }^{2}-2{x}_{\text{A}}\rho u}{1-{u}^{2}}}.$$

As above, this distribution is normalised such that the integral of d*n*(*ρ*) over all distances (0 to 2*R*) is unity.

As before, we find it instructive to look at this result in the limits. Thus, to first order, we find

7 $$\text{d}n\left(\rho \right)=\frac{9}{{4R}^{2}}\rho \mathrm{d}\rho +\dots .$$

Compared to a circle or a cylinder (Eq. 3), we then have a moderate increase of the pre-factor, from 2 to 9/4. If we now consider the co-localisation, in the projected geometry, of two objects within the sphere we find that it is given by (9/8) (*ξ*/*R*)^2^. That is, the probability that two objects placed at random within the sphere are *ξ* apart in projected coordinates is now different compared to our naive expectation based on overlap of areas [(*ξ*/*R*)^2^]. Nevertheless, the increased probability of co-localisation is still rather small, increasing by just a factor of 9/8 = 1.25.

Hence, we found that the naive idea of just considering overlap is (to first order) exactly correct for a circle, exact also for projected distances for any object that is uniform in the third dimension and only moderately modulated for a sphere. We may, therefore, hypothesize that the effect of projection will often be rather small, at least to leading order.

### Co-localisation of arbitrary number of objects

So far, we have given a quantitative discussion of how geometry affects the distribution of distances when objects distribute by chance completely uniformly within a space of a certain shape. The distribution of distances is not the same as the probability of co-localisation, but may readily be phrased (by performing an integration) as the co-localisation between two (and two only) objects. When several (i.e., more than two) objects distribute within the space, however, combinatorial effects must also be considered.

Thus, let the space contain *N*_A_ objects of type A and *N*_B_ objects of type B. We will assume that the probability of any given pair of objects being co-localised can be found from the distribution of distances (previous sections). This is not exactly true, because how close two given objects are is not independent of how close one of those objects is to all of the other objects. It is, however, a first approximation and a good one for a rather small number of objects.

Let us now consider the probability, *P*_*n*_, that *n* of the *N*_A_ objects of type A are co-localised with either of the objects of type B, that is, that *n* objects of type A are within a certain distance of any of the B objects. We consider the case that it is irrelevant whether an object of type A is co-localised with just one or several of the B objects (as long as it is, indeed, co-localised with at least *one*). One can then show (see Online Resource 1, “Co-localisation of arbitrary number of objects” section) that *P*_*n*_ is given by

8 $${P}_{n}=\left(\begin{array}{c}{N}_{\mathrm{A}}\\ n\end{array}\right){\left(1-{\left(1-x\right)}^{{N}_{\mathrm{B}}}\right)}^{n}{\left(1-x\right)}^{{N}_{\mathrm{B}}\left({N}_{\mathrm{A}}-n\right)},$$

where *x* is the probability that one object of type A and one of type B is within a given distance, *ξ*. As above, we may find *x* by integration of the distribution of distances (Eq. 1)*.* Expression (8) can be confirmed to be normalised such that ${\sum }_{n=1}^{{N}_{\mathrm{A}}}{P}_{n}$ = 1 (see Online Resource 1 for the argument). Experimental measurements are typically not phrased in terms of probabilities; a useful experiment–theory link would seem to be the average number of co-localised A objects, ${\sum }_{n=0}^{{N}_{\mathrm{A}}}{nP}_{n}.$ One can show (again, see Online Resource 1 for a complete derivation) that this is given by

9 $${\sum }_{n=0}^{{N}_{\mathrm{A}}}{nP}_{n}={N}_{\mathrm{A}}\left(1-{\left(1-x\right)}^{{N}_{\mathrm{B}}}\right).$$

If we then are interested in the proportion of co-localised A objects, we should then simply divide by the total number of A objects, *N*_A_. Overall, we then have that the average co-localisation is simply

10 $$\left(1-{\left(1-x\right)}^{{N}_{\mathrm{B}}}\right).$$

Though it is implicitly in Eqs. (8)–(10) it is perhaps nevertheless worth reiterating what parameters enter our co-localisation calculation. First, it of course depends on what inter-object distance we consider two objects to be co-localised within. We have called this parameter *ξ* and it signifies the limited resolution with which we can resolve objects. A useful value for this parameter is hence of the order of the diffraction limit (for conventional, not super-resolution, imaging approaches). Furthermore, the co-localisation depends on the geometry and size of the space (cell) we consider. Importantly, the co-localisation also depends on the number of objects within this space; at least in our approximation, however, it only depends on the number objects of type B.

### Exemplification of results for bacterial cells

To exemplify the discussion, we use realistic parameters and shapes for bacterial cells. We start by considering a spherical cell, which we assume has been studied using confocal microscopy in a single plane, so that only an optical slice, rather than the full cell, has been imaged. To take into account that this situation is geometrically more complicated than those analysed theoretically above, we performed numerical simulations to calculate the distribution of distances (see “Methods” section). The actual co-localisation was, however, calculated analytically using the theory above with the simulated distance distribution as input. We assume that the optical slice has been taken in the centre of the cell and that its thickness is 0.3 μm; likely, the results are not very sensitive to this numerical choice (see above). As an example of a rather spherical cell we consider *L. lactis*, with a diameter in the range of 0.5–1.5 μm. Furthermore, we show results for several different distances, *ξ*, used to define when two objects are co-localised, namely for *ξ* = 0.1, 0.2 and 0.3 μm. All these are below or of the order of the diffraction limit for typical optical wavelengths, though 0.3 μm is nevertheless perhaps on the upper edge of what one may use in practice considering the small size of the cell. The co-localisation expected due to chance also depends strongly on the number of objects (of type B); for definiteness, we will assume that there are five objects of type A, and vary the number of objects of type B.

**Fig. 2:**
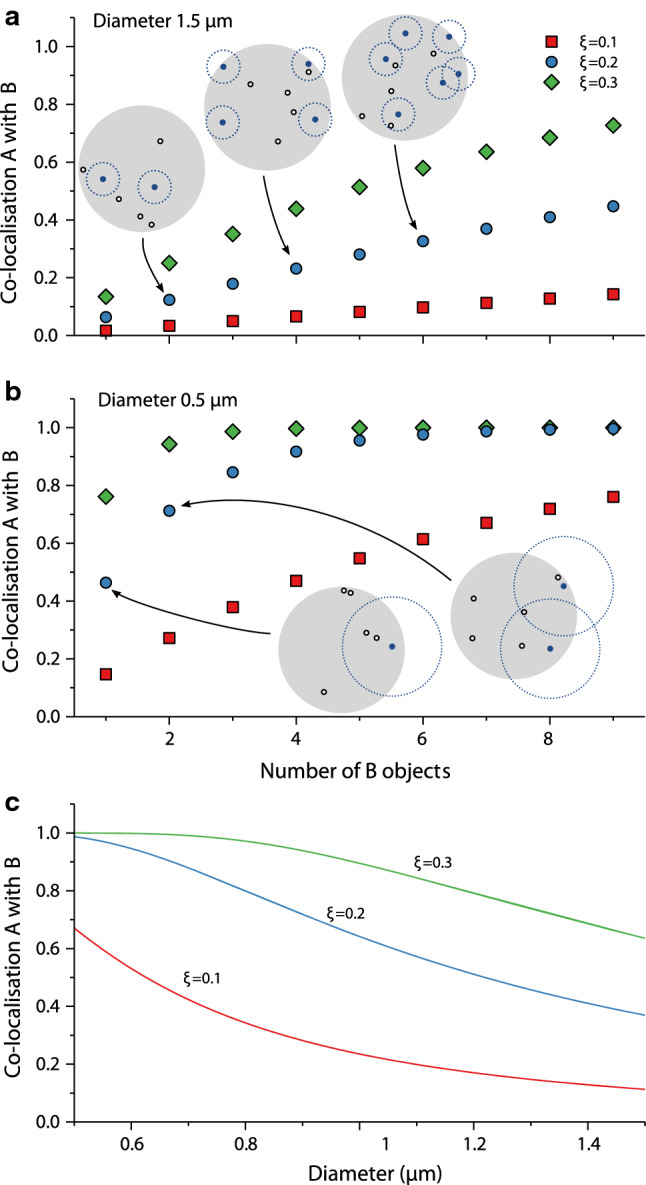
Co-localisation expected due to chance for a spherical cell, considering only the central slice of the cell. The results are presented in terms of the co-localisation of objects of type A with objects of type B. **a** Cell of diameter 1.5 µm, with five objects of type A, varying the number of objects type B. (Images) Sample configurations from simulations corresponding to the (average) co-localisations indicated by arrows. Only the two-dimensional projections are shown. The cell is depicted as a grey outline, while objects of type A and B are indicated in black and blue, respectively. Circles indicate the distance within which an object of type A would be considered co-localised with an object of type B. **b** Same as in panel a, but for a cell with a smaller diameter of 0.5 µm. *N.B.* the images showing sample configurations have been kept at the same size as in panel a for legibility, though in actuality their radii are one third of those in panel **a**. **c** Dependence on the diameter of the cell for five objects of type A and seven objects of type B. *ξ* denotes the inter-object distance used to define when two objects are co-localised and the thickness of the slice was set to 0.3 µm

Figure 2a shows the co-localisation of objects of type A with objects of type B for the larger diameter of 1.5 μm, as a function of the number of objects of type B. It is clear that a rather substantial co-localisation is expected, purely due to chance, already for a moderate number of B objects, at least for the longer distances used to define co-localised (*ξ* = 0.2 and 0.3 μm). Naturally, the situation is even more pronounced for the smaller diameter of 0.5 μm, as shown in Fig. 2b, where the co-localisation expected due to chance is high also for the smaller distance used to define co-localised (*ξ* = 0.1) and already for a few B objects. We may also illustrate the dependence on the diameter; Fig. 2c shows the co-localisation between, for definiteness, five objects of type A and seven objects of type B for varying diameter. As expected, we observe a larger co-localisation the smaller the cell is.

As another example, we consider *E. coli* cells, whose shape we may approximate with that of a cylinder with spherical caps. The diameter is around 0.8–1.2 μm, while the length varies considerably (Phillips et al. 2009). We use similar parameters as above, viz*.*, an optical slice thickness of 0.3 μm; co-localisation distances of *ξ* = 0.1, 0.2 and 0.3 μm; and five objects of type A.

**Fig. 3:**
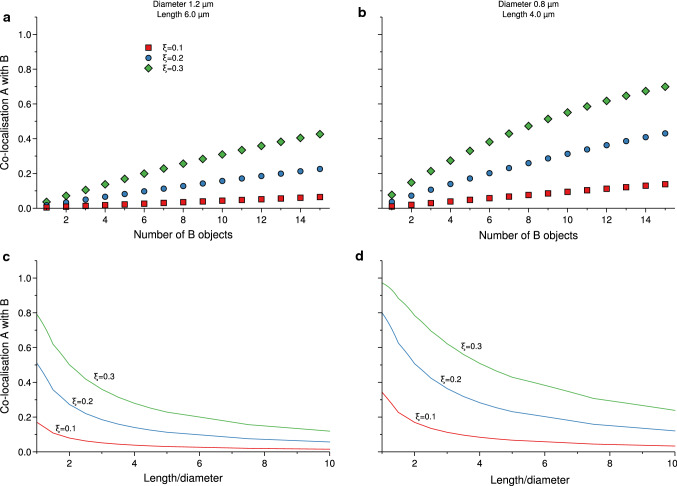
Co-localisation expected due to chance for a cylindrical cell with spherical caps, considering only the central slice of the cell. The results are presented in terms of the co-localisation of objects of type A with objects of type B. **a** Cell of diameter 1.2 µm and length 6.0 µm, with five objects of type A, varying the number of objects of type B. **b** Same as in panel a, but with a smaller diameter of 0.8 µm and a shorter length of 4.0 µm (same ratio of length to diameter as in panel **a**). **c** Dependence on the length of cell for a cell of diameter 1.2 µm containing five objects of type A and seven objects of type B. **d** Same as in panel **a**, but with a smaller diameter of 0.8 µm. *ξ* denotes the inter-object distance used to define when two objects are co-localised and the thickness of the slice was set to 0.3 µm

Figure 3a shows the co-localisation as a function of the number of B objects for a diameter of 1.2 μm and a length (total length, including the length of the cylinder *and* the spherical caps) of the cell five times larger than the diameter; Fig. 3b shows the same, but for a smaller diameter of 0.8 μm. The general conclusion is similar to the case of *L. lactis* above, though with the larger size the co-localisation expected due to chance is smaller for a given number of objects. On the other hand, it is perhaps often the case that the number of objects in the bigger cell type is also larger, so in practice the effects may be rather similar.

Figure 3c, d shows the co-localisation as a function of the length of the cell for five A objects and seven B objects, for both cell diameters used above (1.2 and 0.8 μm, respectively). The results for a length equal to the diameter reproduces the results expected for a sphere (cf. Fig. 2c), while for larger lengths, the co-localisation expected due to chance naturally becomes smaller and smaller. As expected, the co-localisation is higher in the smaller cell (Fig. 3d) compared to the larger (Fig. 3c), keeping the number of objects the same.

## Conclusions

This work has considered the co-localisation that may be expected, purely due to chance, between two types of objects when the two objects remain within a certain space. We argue that placing the objects completely at random within the space is the most natural way of viewing ‘purely due to chance’. Nevertheless, one must acknowledge that in certain situations, where more is known with regards to how the objects distribute, then a different ‘baseline’ may be more useful. For example, in cells with a cytoskeleton and for objects that remain attached to the cytoskeleton, clearly a random placement over the cytoskeleton would be more appropriate.

To gain theoretical understanding, we based our approach on the distribution of distances within the space. We could thereby find analytical expressions, at least for certain simple geometries, such as the circle, the cylinder in projected coordinates, the sphere in projected coordinates and [in a previous work (Kelly et al. 2015)] the surface of the sphere in projected coordinates. Based on these analytical expressions, we could show how the effect of the confined geometry changes the distribution of distances compared to infinite space. We could also show that the effect of a projected geometry is typically rather small, at least for the spaces we considered.

To calculate something closer to the co-localisation measured and reported in experimental studies, we next used the distribution of distances within a space as a basis and considered the combinatorial effect of placing a given number of objects within that space. The formulation based on the distribution of distances implicitly disregards what we may call many-body effects, that is, that the probability that two objects are at a given distance is not independent of all other objects within the space. Nevertheless, it is a good approximation as long as the number objects is fairly modest, which seems like the situation of most practical interest for co-localisation studies. Using this approximation, we could thereby write down analytical expression for the co-localisation expected due to chance.

To illustrate the theory and exemplify the magnitude of the effect, we then considered two models of bacterial cells: one spherical, to represent *L. lactis*; and one cylindrical with caps, to represent *E. coli*. We assumed these to be investigated by confocal microscopy, so that only a slice of the centre of the cells would be known. To take into account the more complicated geometry, we used numerical simulations to find the distribution of distances, but otherwise used our co-localisation theory for the results. The general conclusion is that the co-localisation expected due to chance can be rather substantial and particularly so for smaller cells and larger number of objects.

In previous studies published by the Robinson group, co-localisation between pairs of DNA replication and repair enzymes were measured in rod-shaped *E. coli* cells (diameter ~ 1.2 μm, length ~ 5 μm; Robinson et al. 2015; Henrikus et al. 2018). In these studies, chance co-localisation for species A against species B was estimated by simply calculating the fractional area of the cell occupied by species B foci and their search radii (*ξ*). The results presented in Fig. 3a show a near-linear relationship between chance co-localisation and the number of B objects for cylindrical cells with dimensions that approximate those of *E. coli* cells. This indicates that the simple area-based method we used to estimate chance co-localisation in our previous studies is actually quite reasonable. Nevertheless, it is useful to have the results presented here to support that conclusion. Importantly, a simpler co-localisation measure would not be suitable for spherical cells (Fig. 2a, b), or for cylindrical cells of smaller dimensions (Fig. 3b), where the relationship between chance co-localisation and the number of B objects is more complex. The mathematical framework presented in the current study provides a means to calculate chance co-localisation in these smaller cell types.

Overall, chance co-localisation can be substantial and the co-localisation that one observes in microscopy must be interpreted with reasonable models for chance co-localisation in hand.

## Methods

**Fig. 4:**
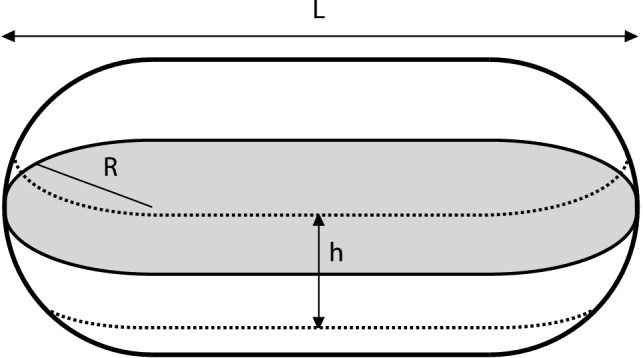
Slice through cylinder with spherical caps. The slice is taken symmetrically through the cell and distances calculated in the projected plane (grey). *L* denotes the total length of the shape, which includes the spherical caps; *R* the radius of each of the two spherical caps; and *h* the thickness of the slice

The results for a sphere (Fig. 2) and cylinder with caps (Fig. 3), both evaluated only for a slice through the centre, were based on numerical simulations for the distribution of distances, but otherwise, the analytical theory for co-localisation discussed above was used. We considered the geometry shown in Fig. 4; compared to this figure, the results for a sphere is simply given by the total length being 2*R* so that only the caps remain.

To calculate the distribution of distances numerically by simulations, a number of objects were positioned randomly in a uniform and independent fashion within the geometrical body, the distribution of distances calculated and the procedure repeated until the average distribution was smooth. Since performing the simulations is computationally expensive, we only did so once. Thus, simulations were performed for a range of different lengths of the cylinder and thicknesses of the slice. A second-order polynomial was subsequently fitted to the distance distribution, and a table of coefficients as a function of cylinder length and slice thickness built up. Thereby, a computationally inexpensive calculation of the distance distribution could be achieved by interpolation within the table of polynomial coefficients.

## Supplementary Information

Below is the link to the electronic supplementary material.

Supplementary file1 (PDF 340 KB) Detailed mathematical derivations and arguments of the results presented in the main text.

## Data Availability

All relevant data are within the manuscript.
