# Supplementary material for: Single-molecule localisation microscopy: accounting for chance co-localisation between foci in bacterial cells
Source: Eur Biophys J. 2021 Jun 19;50(7):941–50. doi: 10.1007/s00249-021-01555-z (PMC8448688; doi:10.1007/s00249-021-01555-z)
Supplement: Supplementary file 1 — Supplementary file1 (PDF 340 KB) Detailed mathematical derivations and arguments of the results presented in the main text. [file 249_2021_1555_MOESM1_ESM.pdf]

European Biophysics Journal

Online Resource 1 to

Single-Molecule Localisation Microscopy: Accounting for Chance Co-Localisation Between Foci in Bacterial Cells

Christoffer Åberg[a,*] & Andrew Robinson[b,c]

[a] Groningen Research Institute of Pharmacy, University of Groningen, Antonius Deusinglaan 1, 9713AV Groningen, The Netherlands

[b] Molecular Horizons Institute and School of Chemistry and Molecular Bioscience, University of Wollongong, Wollongong, NSW 2522, Australia

[c] Illawarra Health and Medical Research Institute, Wollongong, NSW 2522, Australia

[*] Corresponding author (christoffer.aberg@rug.nl)

Derivation of Distribution of Distances within a Circle

For objects that distribute uniformly and independently within the circle, we can calculate the distribution of inter-object distances, ρ, in essence by positioning two points, *A* and *B*, within the circle, *A* anywhere and *B* at a distance ρ from *A*, and integrating over all possible positions for *A* and *B*. The calculation is facilitated by using the high amount of symmetry, but also by explicitly including the quantity of interest – that is, the inter-object distance, ρ – into the derivation.

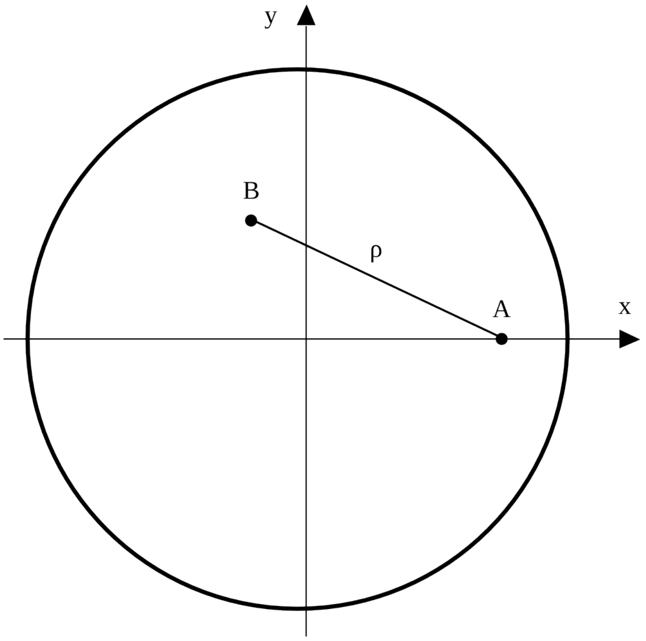

Figure 1 Choice of the points *A* and *B* to calculate the distribution of distances within a circle. Point *A* is chosen to lie on the positive *x* axis, while point *B* is chosen to lie in the upper half plane.

We may, without loss of generality, choose point *A* to lie on the positive *x* axis, and point *B* to lie in the upper half plane (Fig. 1). Parametrising the position of point *A* in polar coordinates we have

$$\boldsymbol{r_A}=(x_A\cos\theta, x_A\sin\theta)$$

where $x_A\in[0,R]$, $\theta\in[0,2\pi]$ and R is the radius of the circle. Integrating out the θ dependence to account for our choice of *A*, we then have that the area element we need for integrating over all positions of *A* is given by $dA_A=2\pi x_A\,dx_A$ which should only be integrated over x_A.

For point *B* we choose a parametrisation explicitly based upon its distance, ρ, to point *A*. Thus we write

$$\boldsymbol{r_B}=(x_A+\rho\cos\theta, \rho\sin\theta)$$

where for a given x_A $\rho\in[0,R+x_A]$. The limits on θ depend on ρ (see Fig. 2): for $\rho\in[0,R-x_A]$ $\theta\in[0,2\pi]$, while for $\rho\in[R-x_A,R+x_A]$ $\theta\in[\theta_c(x_A,\rho),\pi]$, where

$$\cos\theta_c=\frac{R^2-x_A^2-\rho^2}{2x_A\rho}$$

The area element is then given by $dA_B=2\rho\,d\rho\,d\theta$, where the factor of 2 accounts for our choice of *B*. In order to calculate the distribution of distances we, however, need the integration limits for a given ρ, rather than for a given x_A. Thus, $x_A\in[max(R-\rho,\rho-R),R]$ and $\theta\in[\theta_c(x_A,\rho),\pi]$. Additionally, if $\rho\in[0,R]$ the integration should *also* be carried out over the region defined by $x_A\in[0,R-\rho]$ and $\theta\in[0,\pi]$.

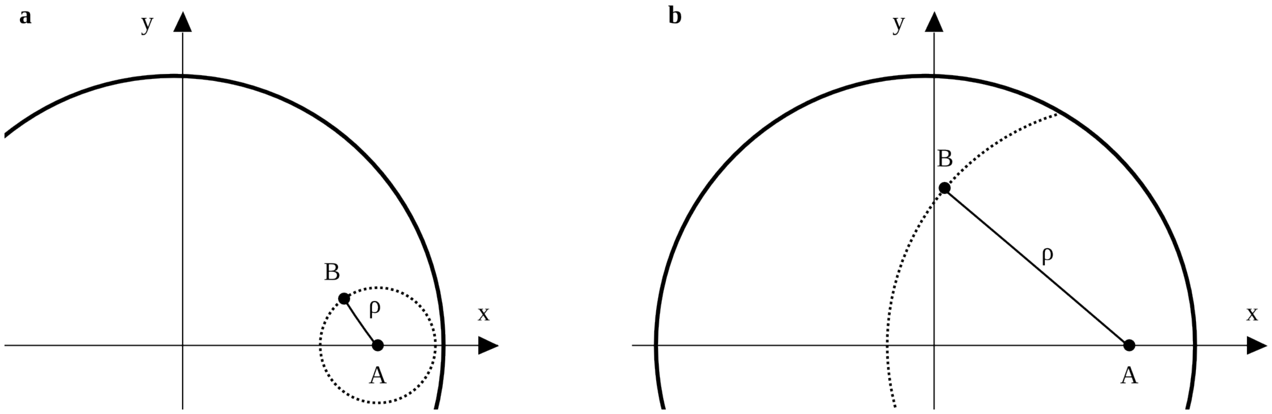

Figure 2 Integration limits to calculate distribution of distance for circle. a, For smaller ρ all points *B* on the circle (dotted) of radius ρ centered on the point *A* lie within the large circle (solid). **b,** for larger ρ, on the other hand, not all points *B* on the circle (dotted) of radius ρ centered on the point *A* lie within the large circle (solid). The limits on the angle can be found from the condition that the smallest angle is such that the circle (dotted) of radius ρ centered on the point *A* intersect with the large circle (solid).

The distribution of distances is then given by

$$rect_{[0,R]}(\rho)\int_{x_A=0}^{x_A=R-\rho}\int_{\theta=0}^{\theta=\pi}2\pi x_A dx_A 2\rho d\rho d\theta+\int_{x_A=max(R-\rho,\rho-R)}^{x_A=R}\int_{\theta=\theta_c}^{\theta=\pi}2\pi x_A dx_A 2\rho d\rho d\theta=$$

$$=2\pi^2\rho d\rho\left(R^2-rect_{[R,2R]}(R-\rho)^2-\frac{2}{\pi}\int_{x_A=max(R-\rho,\rho-R)}^{x_A=R}x_A\theta_c dx_A\right)$$

So far we have not considered normalisation; it is, however, clear that the distribution thus constructed is normalised such that an integration over ρ from 0 to 2R will give the area of the circle squared. A distribution normalised to unity can therefore be found by dividing the above result with $(\pi R^2)^2$.

In summary, the normalised distribution of distances is then given by

$$\mathrm{d}n(\rho)=\frac{2}{R^4}\rho\mathrm{d}\rho\left(R^2-\mathrm{rect}_{[R,2R]}(\rho)(R-\rho)^2-\frac{2}{\pi}\int_{x_A=\max(R-\rho,\rho-R)}^{x_A=R}x_A\arccos\left(\frac{R^2-x_A^2-\rho^2}{2x_A\rho}\right)\mathrm{d}x_A\right)$$

where R is the radius of the circle. This distribution has been normalised such that the integral of dn(ρ) is unity.

An expansion to first order in ρ gives the result

$$dn(\rho)=\frac{2}{R^2}\rho\mathrm{d}\rho+\cdots.$$

We can compare this result to those used in statistical mechanics. In three dimensions an ideal gas has $\mathrm{d}n(r)=\rho 4\pi r^2\mathrm{d}r$ where ρ here is the density in number of particles per unit volume and r is distance.[17] In analogy with this, an ideal gas in two dimensions has $\mathrm{d}n(r)=\sigma 2\pi r\mathrm{d}r$, where σ is the number of particles per unit area. With our choice of normalisation $\sigma=(\pi R^2)^{-1}$ and the same result as above follows.

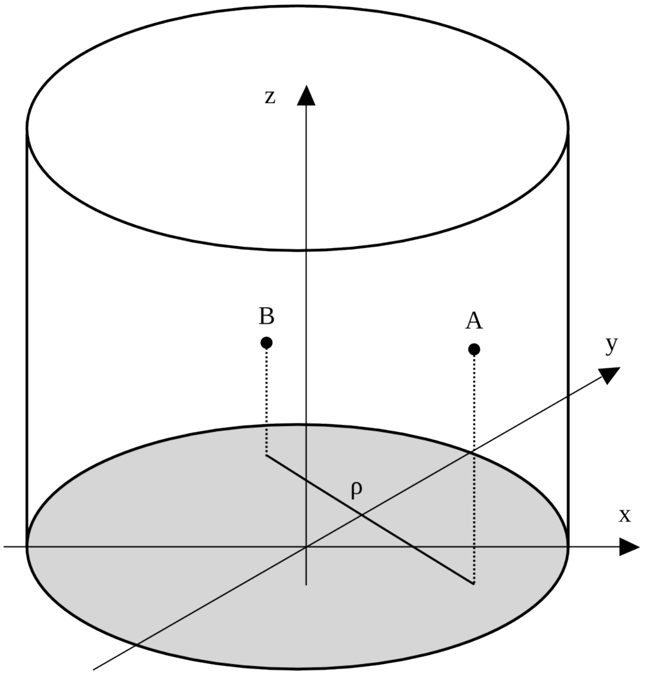

Figure 3 Cylinder and the projected distance between two points, *A* and *B*.

Distribution of Distances within Cylinder in Projected Coordinates

Consider a cylinder within which objects distribute uniformly and independently, but where we are only able to measure distances in the plane perpendicular to the cylinder axis (Fig. 3). One can then argue that the projected distance distribution is exactly the same as for a circle (above). One readily arrives at this conclusion mathematically, simply by parameterising the points *A* and *B* in the same way as for the circle in the *x* and *y* directions. Since the integration intervals in the *z* direction do not depend on the other directions, these parts can be readily integrated and cancel with the corresponding factors when normalising. All the results for a circle then remains true also for a cylinder, as long as the distances are interpreted as projected ones.

Derivation of Distribution of Distances within Sphere in Projected Coordinates

For objects that distribute uniformly and independently within the sphere, we can calculate the distribution of inter-object distances projected in the equatorial plane, ρ, in essence by positioning two points, *A* and *B*, within the sphere, *A* anywhere and *B* such that the distance between *A* and *B* projected in the equatorial plane is ρ, and integrating over all possible positions for *A* and *B*. The derivation proceeds similarly to the circle (above) and is again facilitated by symmetry.

Thus we may, again without loss of generality, choose point *A* to lie in the upper hemisphere and to have its projected position on the positive *x* axis, and point *B* to lie in the upper hemisphere and with positive *y* (Figure 4). Parametrising the position of point *A* in cylindrical coordinates we have

$$\boldsymbol{r}_A=(x_A\cos\theta, x_A\sin\theta, z)$$

where $x_A\in[0,R]$, $\theta\in[0,2\pi]$, $z\in\left[-\sqrt{R^2-x_A^2},\sqrt{R^2-x_A^2}\right]$ and *R* is the radius of the sphere. Integrating out the θ and z dependence to account for our choice of *A*, we then have that the volume element we need for integrating over all positions of *A* is given by $dV_A=4\pi\sqrt{R^2-x_A^2}\,x_A\,dx_A$ which should only be integrated over x_A.

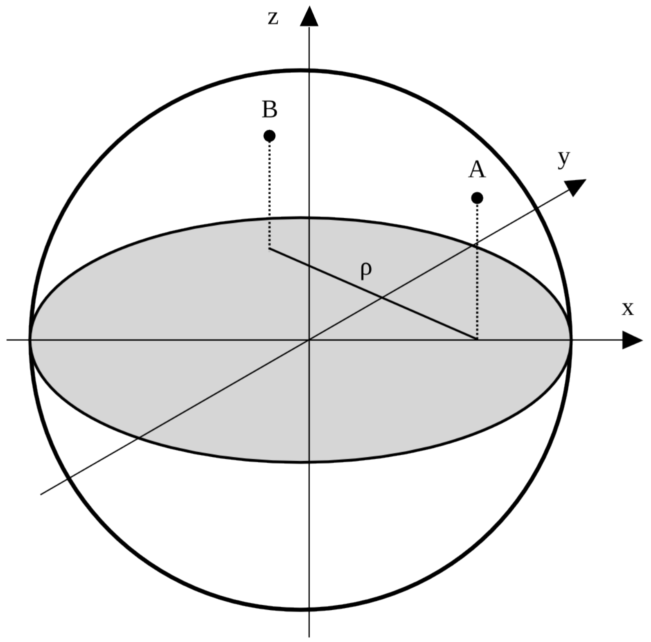

Figure 4 Choice of the points *A* and *B* to calculate the distribution of distances within sphere in projected coordinates. Point *A* is chosen to lie in the upper hemisphere and such that its projection (dotted line) into the equatorial plane (grey) is on the positive *x* axis. Point *B* is chosen in the upper hemisphere and with a positive *y* coordinate, that is, on the hemisphere furthest away. The schematic also shows the distance, ρ, between the positions of point *A* and *B* projected into the equatorial plane.

For point *B* we again choose a parameterisation explicitly based upon its projected distance, $\rho,$ to point *A*. Thus we write

$$\boldsymbol{r_B}=(x_A+\rho\cos\theta,\rho\sin\theta,z)$$

where the limits in terms of ρ and θ for fixed x_A are the same as above. The limits on z can be shown to be $z\in\left[0,\sqrt{R^2-x_A^2-\rho^2-2x_A\rho\cos\theta}\right]$. The volume element is then $dV_B=4\rho d\rho d\theta dz,$ where the factor 4 accounts for our choice of *B*. Integrating out the z dependence we find $dV_B=4\rho d\rho\sqrt{R^2-x_A^2-\rho^2-2x_A\rho\cos\theta}\,d\theta$.

The re-writing of the integration regions proceeds as in the case of a circle (above) and we thus have that the distribution of distances is given by

$$rect_{[0,R]}(\rho)\int_{x_A=0}^{x_A=R-\rho}\int_{\theta=0}^{\theta=\pi}I(x_A,\theta;\rho)d\theta dx_A+\int_{x_A=max(R-\rho,\rho-R)}^{x_A=R}\int_{\theta=\theta_c}^{\theta=\pi}I(x_A,\theta;\rho)d\theta dx_A$$

where we have used the short-hand $I(x_A,\theta;\rho)=4\pi\sqrt{R^2-x_A^2}x_A 4\rho d\rho\sqrt{R^2-x_A^2-\rho^2-2x_A\rho\cos\theta}$ for brevity. Again, we observe that by construction the distribution thus obtained is normalised such that its integral over ρ from 0 to 2*R* is equal to the volume of the sphere squared; consequently we must divide by $(4\pi/3R^3)^2$ if want the distribution normalised to unity.

In summary, the distribution of distances is thus given by

$$\mathrm{d}n(\rho)=\frac{9}{\pi R^6}\rho\mathrm{d}\rho\left(\mathrm{rect}_{[0,R]}(\rho)\int_{x_A=0}^{x_A=R-\rho}\int_{u=-1}^{u=1}f(\rho,x_A,u)\mathrm{d}x_A\mathrm{d}u+\int_{x_A=\max(R-\rho,\rho-R)}^{x_A=R}\int_{u=-1}^{u=(R^2-x_A^2-\rho^2)/2x_A\rho}f(\rho,x_A,u)\mathrm{d}x_A\mathrm{d}u\right)$$

in terms of the integrand

$$f(\rho, x_A, u) = \sqrt{R^2 - x_A^2} x_A \sqrt{\frac{R^2 - x_A^2 - \rho^2 - 2x_A \rho u}{1 - u^2}}$$

(we have here also re-written the integration over θ into one over u=cos θ). This distribution has been normalised such that the integral of dn(ρ) is unity.

Co-Localisation of Arbitrary Number of Objects

We consider a space that contains N_A objects of type *A* and N_B objects of type *B* and work under the assumption that the probability that a given pair of objects are co-localised can be found from the distribution of distances. We, furthermore, consider the probability, P_n, that *n* of the N_A objects of type *A* are co-localised with either of the objects of type *B*, that is, that *n* objects of type *A* are within a certain distance of any of the *B* objects. There are $\binom{N_A}{n}$ possibilities of drawing the *n* objects from a total number of N_A objects of type *A*. The probability that it is co-localised with exactly *k* of the N_B objects of type *B* is then

$$\binom{N_B}{k} x^k (1-x)^{N_B-k}$$

where *x* is the probability that the distance between two objects is within a given distance. Here the first factor originates from the different possibilities of choosing *k* objects of the N_B objects of type *B* possible; the second factor is the probability that *k* of those objects are within the distance; and the final factor is the probability that the remaining N_B - *k* objects are *not* within the given distance. We consider the case that it is irrelevant whether an object of type *A* is co-localised with just one or several of the *B* objects (as long as it is, indeed, co-localised with at least *one*). Then we must sum all possibilities to arrive at

$$\sum_{k=1}^{N_B} \binom{N_B}{k} x^k (1-x)^{N_B-k} = 1-(1-x)^{N_B}$$

where the last equality follows from the binomial theorem. We were, however, interested in *n* co-localisations, and hence find

$$\left(1-(1-x)^{N_B}\right)^n$$

The remaining N_A - *n* of the *A* objects are not co-localised; for each of these objects the only possibility is that it is not within the given distance of *any* of the N_B objects of type *B*. The probability of this happening is $(1-x)^{N_B}$ and since there are N_A - *n* objects we find

$$\left((1-x)^{N_B}\right)^{N_A-n}$$

Putting everything together, we then have that the probability of having *n* objects of type A co-localised with type *B* is

$$P_n = \binom{N_A}{n} \left(1-(1-x)^{N_B}\right)^n (1-x)^{N_B(N_A-n)}.$$

We observe that this is the *n*th coefficient in a binomial expansion of

$$\left(\left(1-(1-x)^{N_B}\right)+(1-x)^{N_B}\right)^{N_A}$$

which, in fact, is just unity, written in a complicated fashion; the sum of all probabilities, $\sum_{n=1}^{N_A} P_n$, is then simply this expression, so the probability is then clearly normalised correctly.

To find the average co-localisation, that is, the average number of co-localised *A* objects, we must calculate $\sum_{n=0}^{N_A} nP_n$. To this end, one may ntroduce the generating function

$$g(z) \equiv \sum_{n=0}^{N_A} z^n P_n = \sum_{n=0}^{N_A} \binom{N_A}{n} z^n \left(1-(1-x)^{N_B}\right)^n (1-x)^{N_B(N_A-n)} = \left(\left(1-(1-x)^{N_B}\right) z + (1-x)^{N_B}\right)^{N_A}$$

where the final equality follows from the binomial theorem. In terms of this generating function we have that the average number of co-localised objects is $\sum_{n=0}^{N_A} nP_n = g'(z=1)$, and we find

$$\sum_{n=0}^{N_A} nP_n = N_A\left(1-(1-x)^{N_B}\right)$$

and hence this is the average number of co-localised objects.
